# Dosing Cariprazine Within and Beyond Clinical Trials: Recommendations for the Treatment of Schizophrenia

**DOI:** 10.3389/fpsyt.2021.770234

**Published:** 2022-01-05

**Authors:** Elmars Rancans, Zsófia Borbála Dombi, Ágota Barabássy

**Affiliations:** ^1^Department of Psychiatry and Narcology, Riga Stradins University, Riga, Latvia; ^2^Gedeon Richter Plc., Medical Division, Budapest, Hungary; ^3^Department of Psychiatry, University of Oxford, Oxford, United Kingdom

**Keywords:** cariprazine, schizophrenia, antipsychotic, dosing, psychopharmacotherapy

## Abstract

Although the optimal dosing of an antipsychotic medication is known to be essential in the long-term management of schizophrenia, in case of novel drugs such as cariprazine, determining the right dosing strategy is not that simple. Without decades of experience with a particular compound, evidence regarding dosing and titration comes primarily from double-blind, placebo controlled clinical trials that are not necessarily mirroring the real-life experiences of doctors. Via summarizing data from both clinical data (*n* = 3275) and real-world evidence (observational study *n* = 116, case studies *n* = 29), this perspective paper aims to shed a light on the appropriate dosing strategies of cariprazine from treatment initiation through switching strategies to concomitant medications.

## Introduction

Antipsychotic medication, has been prescribed as the first line of treatment in schizophrenia since the 1950s (1, 2). While the so-called typical or first-generation antipsychotics (FGAs) such as haloperidol have been associated with considerable side effects, atypical or second-generation antipsychotics (SGAs) changed the view of psychosis treatment by offering similar level of efficacy as FGAs but with much lower rates and severity of adverse events (3). Throughout the past few decades however, third-generation antipsychotics (TGAs), have been in the spotlight given their ability to improve not only positive but potentially negative and cognitive symptoms as well (4–7). Many of these atypical antipsychotics are characterized by dopamine partial agonism (8), which explains their improved efficacy and safety profile (9) but also the fact why practitioners feel challenging to find the right strategy to dose them (10, 11).

The optimal dosing of antipsychotics is known to be essential in the long-term management of schizophrenia (12). The general rule is that one should aim for a treatment initiation and titration strategy that promotes quick and adequate response without the introduction of side effects that are too bothersome for the patient (12). Indeed, discontinuation and frequent switching between different antipsychotics due to adverse events or insufficient therapeutic response are highly common in schizophrenia patients (13, 14)—many practitioners switch or start polypharmacy before optimizing the current medication dose in order to address the patients' complains and to avoid non-adherence (12).

In case of novel drugs however, determining the right dosing strategy is not that simple. Without decades of experience with a particular compound, evidence regarding dosing and titration comes primarily from clinical trials (12). Aiming to determine efficacy against placebo with the lowest possible side effects, in such studies manufacturers utilize doses that are often much lower than what is actually needed in real life (12, 15). In addition, patients involved in clinical trials are required to fit into a highly rigorous criteria and hence can be immensely different from those seen by doctors in their everyday clinical practice (16). Thus, in this paper, we aim to summarize the clinical data of cariprazine dosing within and beyond clinical trials.

## Methods

Trials, studies, and cases for this perspective were identified by searching Embase and Medline databases for English language articles published in peer-reviewed journals between 1 January 2000 and 1 June with search terms “(cariprazin^*^ OR “rgh-188” OR rgh188) AND (“case report^*^” OR “case stud^*^” OR “case series^*^” OR “trial^*^” OR “stud^*^”).” Searches by hand were also conducted to identify additional relevant articles. Articles were included if they: (1) were an original research conducted with human subjects; (2) involved patients with diagnosis of schizophrenia; (3) provided adequate information regarding the dosing of cariprazine. Out of the 186 findings, 6 clinical trials, 1 observational study and 29 cases met the inclusion criteria.

## Cariprazine, A Third-Generation Antipsychotic Agent

Cariprazine is a TGA that is approved for the treatment of schizophrenia by the Food and Drug Administration (FDA) and the European Medicines Agency (EMA). It is a dopamine D3 receptor preferring partial agonist at the D3/D2 and at the serotonin 5-HT1A receptors and an antagonist at the 5-HT2B receptors (17). Compared to other antipsychotics, cariprazine's uniqueness is based in its high potency for the D3 receptor that is higher than what is exhibited by dopamine itself, resulting in full D3 receptor occupancy at clinically relevant doses (17). There are two major active metabolites of cariprazine, namely desmethyl cariprazine (DCAR) and didesmethyl cariprazine (DDCAR) (18, 19). Both are pharmacologically equipotent to cariprazine and are known to be jointly responsible for the overall therapeutic effect (18, 19).

Throughout the clinical development programme for the treatment of schizophrenia, the efficacy and safety of cariprazine were established in 8 clinical trials; 4 short-term, randomized, double-blind, placebo-controlled studies with acute patients and 4 long-term studies of various design. In the short-term (6-week) studies patients who had current exacerbation of schizophrenia for <2 weeks in duration and were at least moderately ill were included (20–23). Within the 4 long-term studies, there were two 48-week open-label, flexible-dose safety trials, which served as extensions to the short-term studies (24, 25). The efficacy of cariprazine for the prevention of relapse in patients with acute schizophrenia was also evaluated in a long-term (up to 97 weeks) trial with a randomized, double-blind, placebo-controlled design (26). Finally, the last clinical trial, a 26-week, double-blind, active-controlled study, measured the efficacy of cariprazine in predominant negative symptoms (7).

Additionally to the clinical trials, there was one observational study in Latvia involving patients who were experiencing predominant negative symptoms despite receiving antipsychotic medication (27). Furthermore, several cases have been published that discuss cariprazine's effectiveness and safety in various schizophrenia patients.

### Treatment Initiation With Cariprazine

Cariprazine is currently approved in four doses: 1.5, 3.0, 4.5, and 6.0 mg/day (28). According to the Summary of Product Characteristics (SmPC) the recommended starting dose for cariprazine is the lowest available dose, 1.5 mg/day (28, 29). Indeed, as summarized in Table 1, in the majority of cases cariprazine treatment was initiated with this dose. Exceptions were three patient cases where cariprazine was prescribed in the dose of 3.0 mg/day (40), as well as the Latvian observational study where 7.7% of patients received 3.0 mg/day, 3.4% 6.0 mg/day and 1.7% 4.5 mg/day as their starting dose (27). Importantly, as reported in the cases, the higher starting dose was well-tolerated and quick improvement in behavior was detected (40).

**Table 1:** Dosing strategies with cariprazine.

**Author**	**Study type (patient number)**	**Dosing scheme**	**Switching strategy**	**Starting dose**	**Dosing strategy**	**Maintenance dose**	**Concomitant medication**
Amore et al. (30)	Case study (1)	Flexible 1.5–6.0 mg/day	Full-dose overlap from risperidone	1.5 mg/day	3.0 mg/day on day 15	3.0 mg/day	Risperidone gradually discontinued
Aubel (31)	Case study (1)	Flexible 1.5–6.0 mg/day	Risperidone was tapered to 2 × 0.5 mg daily	1.5 mg/day	3.0 mg/day on day 4 and 4.5 mg on day 14	4.5 mg/day	Risperidone gradually discontinued
Aubel (31)	Case study (1)	Flexible 1.5–6.0 mg/day	Cross-titration from clozapine and amisulpride	1.5 mg/day	3.0 mg/day on day 4	4.5 mg/day	-
Aubel (31)	Case study (1)	Flexible 1.5–6.0 mg/day	Aripiprazole 10 mg and risperidone 0.5 mg were discontinued	1.5 mg/day	3.0 mg/day on day 2 and 4.5 mg/day on day 3	4.5 mg/day	-
De Berardis et al. (32)	Case study (1)	Flexible 1.5–6.0 mg/day	Cariprazine as add-on	1.5 mg/day	3.0 mg/day on day 8	3.0 mg/day	Clozapine
De Berardis et al. (32)	Case study (1)	Flexible 1.5–6.0 mg/day	Cariprazine as add-on	1.5 mg/day	3.0 mg/day on day 22	3.0 mg/day	Clozapine
De Berardis et al. (33)	Case study (1)	Flexible 1.5–6.0 mg/day	No previous treatment	1.5 mg/day	3.0 mg/day on day 4, 4.5 mg/day on day 30	4.5 mg/day	-
De Berardis et al. (33)	Case study (1)	Flexible 1.5–6.0 mg/day	No previous treatment	1.5 mg/day	3.0 mg/day after a few days, 4.5 mg/day and then 6.0 mg/day after 14 days	6.0 mg/day	Alprazolam
Di Sciascio et al. (34)	Case study (1)	Flexible 1.5–6.0 mg/day	Cross-titration from risperidone over 2 days	1.5 mg/day	3.0 mg/day on day 2	3.0 mg/day	Risperidone discontinued
Di Sciascio et al. (34)	Case study (1)	Flexible 1.5–6.0 mg/day	Cross-titration from olanzapine over 2 weeks	1.5 mg/day	6.0 mg/day	6.0 mg/day	Olanzapine gradually discontinued by day 15
Durgam et al. (23)	Phase II/III clinical study (390)	Flexible 1.5–4.5 mg/day or 6.0–12.0 mg/day	7-day wash-out	1.5 mg/day	1.5–4.5 mg/day group: 3.0 mg/day on day 3, maximum 4.5 mg/day on day 5 6.0–12.0 mg/day group: 3.0 mg/day on day 3, 6.0 mg/day on day 5, maximum 9.0 mg/day on day 7 or 12.0 mg/day by day 9	-	Lorazepam Zolpidem, zaleplon, chloral hydrate, eszopiclone, diphenhydramine, benztropine, propranolol
Durgam et al. (21)	Phase II/III clinical study (675)	Fixed 1.5 mg/day, 3.0 mg/day, 4.5 mg/day	7-day wash-out	1.5 mg/day	If target dose higher than 1.5 mg/day then 3.0 mg/day on day 2, 4.5 mg/day on day 3	1.5 mg/day, 3.0 mg/day, 4.5 mg/day	Lorazepam Zolpidem, zaleplon, chloral hydrate, eszopiclone, diphenhydramine, benztropine, propranolol
Durgam et al. (20)	Phase II/III clinical study (600)	Fixed 3.0 mg/day, 6.0 mg/day	7-day wash-out	1.5 mg/day	3.0 mg/day on day 2, if target dose higher, then 4.5 mg/day on day 3 and 6.0 mg/day on day 4	3.0 mg/day, 6.0 mg/day	Lorazepam Zolpidem, zaleplon, chloral hydrate, eszopiclone, diphenhydramine, benztropine, propranolol
Durgam et al. (26)	Phase II/III clinical study (700)	Flexible: 3.0–9.0 mg/day Fixed: 3.0, 6.0, or 9.0 mg/day	7-day wash-out	1.5 mg/day	Flexible dose: 3.0 mg/day on day 2, 6.0 mg/day on day 6, 9.0 mg/day on day 10 until day 63 Fixed dose: 3.0, 6.0 or 9.0 mg	3.0 mg/day, 6.0 mg/day, 9.0 mg/day	Lorazepam Zolpidem, zaleplon, chloral hydrate, eszopiclone,
					between day 63 to 147 Fixed-dose double blind: randomized to 3.0. 6.0 or 9.0 mg between day 147 to 644		diphenhydramine, benztropine, propranolol
Carmassi et al. (35)	Case study (1)	Flexible 1.5–6.0 mg/day	Cross-titration from aripiprazole over 10 days	1.5 mg/day	3.0 mg/day on day 5, 4.5 mg/day on day 9, and 6.0 mg/day on day 13	6.0 mg/day,	Aripiprazole gradually discontinued, benzodiazepine
Heck et al. (36)	Case study (1)	Flexible 1.5–6.0 mg/day	Discontinuation of quetiapine before start of cariprazine, then adding quetiapine again	1.5 mg/day	3.0 mg/day on day 5	Cariprazine was reduced to 1.5 mg/day 3 days after the onset of akathisia. Another 2 days later, cariprazine was stopped.	Quetiapine
Heck et al. (36)	Case study (1)	Flexible 1.5–6.0 mg/day	Cariprazine as add-on	1.5 mg/day	3.0 mg/day on day 15	Developed severe Parkinsonism, risperidone treatment was fully stopped, 1.5 mg/day cariprazine was maintained	Risperidone and biperiden
Heck et al. (36)	Case study (1)	Flexible 1.5–6.0 mg/day	No previous treatment	1.5 mg/day	3.0 mg/day on day 8, 4.5 mg/day on day 13	4.5 mg/day	Pipamperone, then olanzapine
Heck et al. (36)	Case study (1)	Flexible 1.5–6.0 mg/day	Cross-titration from amisulpride	1.5 mg/day	3.0 mg/day on day 15, 4.5 mg/day on day 29, and 6.0 mg/day on day 85	6.0 mg/day	-
Kane at al. (22)	Phase II/III clinical study (450)	Fixed/flexible 3.0–6.0 mg/day, 6.0–9.0 mg/day	7-day wash-out	1.5 mg/day	3.0–6.0 mg/day group: 3 mg/day until day 14 if inadequate response 4.5 mg/day on days 14 to 15 and 6.0 mg/day thereafter 6.0–12.0 mg/day group: 3.0 mg/day on days 2–3, 6.0 mg until day 14, if inadequate response 7.5 mg/day on days 14 to 15 and 9.0 mg/day thereafter	-	Lorazepam Zolpidem, zaleplon, chloral hydrate, eszopiclone, diphenhydramine, benztropine, propranolol
Kapulsky et al. (37)	Case study (1)	Flexible 1.5–6.0 mg/day	Abrupt discontinuation of clozapine and gradual titration of cariprazine	-	6.0 mg/day by day 7	Discontinued due to urinary retention	-
Mencacci et al. (38)	Case study (1)	Flexible 1.5–6.0 mg/day	Cross-titration from haloperidol and risperidone over 1 month	-	up to 4.5 mg/day	4.5 mg/day	Haloperidol and risperidone gradually discontinued
Mencacci et al. (38)	Case study (1)	Flexible 1.5–6.0 mg/day	Cross-titration from olanzapine over 3 weeks	-	up to 4.5 mg/day until day 21	4.5 mg/day	Olanzapine gradually discontinued, biperiden, lorazepam, antihistamine
Molnar et al. (39)	Case study (1)	Flexible 1.5–6.0 mg/day	No previous treatment	1.5 mg/day	up to 4.5 mg/day until day 14	3.0 mg/day	-
Montes et al. (40)	Case study (1)	Flexible 1.5–6.0 mg/day	No previous treatment	3.0 mg/day	-	3.0 mg/day	-
Montes et al. (40)	Case study (1)	Flexible 1.5–6.0 mg/day	No previous treatment	3.0 mg/day	6.0 mg/day on day 3	6.0 mg/day	Diazepam
Montes et al. (40)	Case study (1)	Flexible 1.5–6.0 mg/day	Abrupt discontinuation of aripiprazole	3.0 mg/day	6.0 mg/day on day 3	6.0 mg/day	Quetiapine
Müller et al. (41)	Case study (1)	Flexible 1.5–6.0 mg/day	Quetiapine and amisulpride wash-out phase over 4 days	1.5 mg/day	3.0 mg/day on day 5, 4.5 mg/day on day 13	4.5 mg/day	-
Müller et al. (41)	Case study (1)	Flexible 1.5–6.0 mg/day	Cross-titration from olanzapine over 3 days and risperidone over 10 days	1.5 mg/day	3.0 mg/day on day 3, 4.5 mg/day on day 24	4.5 mg/day	Risperidone until 4.5 mg cariprazine
Németh et al. (7)	Phase II/III clinical study (460)	Flexible: 3.0–6.0 mg/day	Cross-titration over 2 weeks	1.5 mg/day	3.0 mg/day on day 7–13, 4.5 mg/day on day 14	3.0 mg/day, 4·5 mg/day, 6.0 mg/day	Trihexyphenidyl Hydrochloride, biperiden, propranolol
Rancans et al. (27)	Observational study (116)	Flexible 1.5–6.0 mg/day	Cross-titration over 2 weeks	1.5, 3.0, 4.5, 6.0 mg/day	Cross-titration until day 14	1.5 mg/day, 3.0 mg/day, 4.5 mg/day, 6.0 mg/day 7.5 mg/day	Anti-EPS medication, antidepressants, benzodiazepines, mood stabilizers
Riedesser et al. (42)	Case study (1)	Flexible 1.5–6.0 mg/day	Cariprazine as add-on	1.5 mg/day	Discontinued after 6 days	-	Clozapine, escitalopram
Riedesser et al. (42)	Case study (1)	Flexible 1.5–6.0 mg/day	Cariprazine as add-on	1.5 mg/day	4.5 mg/day	3.0 mg/day	Amisulpride, hydro-chlorothiazide, amlodipine and ramipril
Riedesser et al. (42)	Case study (1)	Flexible 1.5–6.0 mg/day	Abrupt discontinuation of risperidone and olanzapine 4 days later	1.5 mg/day	3.0 mg/day	Discontinued after 14 days	Pantoprazole
Vita et al. (43)	Case study (1)	Flexible 1.5–6.0 mg/day	Cross-titration from risperidone over 9 day	1.5 mg/day	3.0 mg/day on day 4, 4.5 mg/day on day 8	4.5 mg/day	Risperidone discontinued
Vita et al. (43)	Case study (1)	Flexible 1.5–6.0 mg/day	Abrupt-gradual from paliperidone long-acting	1.5 mg/day	3.0 mg/day on day 4, 4.5 mg/day on day 8, 6.0 mg/day on day 12	6.0 mg/day	Paliperidone discontinued

After initiation of treatment, cariprazine doses are recommended to be increased in 1.5 mg increments up to a maximum of 6.0 mg/day, if necessary (28, 29). In general, there are two main titration strategies—a fast and a slow one. Fast titration involves an increase of 1.5 mg/day each day or every second day until the target dose is achieved, as seen in the short-term clinical trials (20, 21, 23). This or similar strategy was utilized in several cases (31, 33–35, 37, 40, 41, 43), where 3.0 mg/day dose was introduced within less than a week after the beginning of the treatment. In these cases, most of the patients exhibited considerable psychotic symptoms with or without negative symptoms and weight gain problems caused by previous medication. The other—slow—titration strategy has been described in one of the long-term studies, where patients with predominant negative symptoms received cariprazine in a dose of 1.5 mg/day until week 1 and then doses were increased in 1.5 mg increments weekly up until 6.0 mg/day, if it was needed (7). It is also worth to note however, that in this study cross-titration with previous antipsychotic medication was performed in the first two weeks of treatment, whereas in the short-term studies a 7-day wash-out period before cariprazine monotherapy was introduced (20–23). Slow titration strategy was also performed in cases where patients were less psychotic (30, 39) or received cariprazine as add-on treatment (32, 36). As expected, in the Latvian observational study both strategies were present (27); in 34% of the patients, dose was increased every 3rd day, in 28% every 4th day, in 6% every 6th day and in 32% every 7th day.

### Switching From Another Antipsychotic to Cariprazine

In case of cariprazine, switching from another antipsychotic can be beneficial if there has been no or only partial response to positive or negative symptoms (27, 31, 32, 35, 37, 41, 43), if the patient suffers from side effects (27, 34–36, 42) that are less common with cariprazine such as weight gain, hyperprolactinemia, sexual disturbance or sedation (44) or if the patients has been prone to non-adherence or substance abuse (29, 35).

In general, there are four possible ways of switching antipsychotics; abrupt, abrupt-gradual, gradual-gradual (i.e., cross-tapering) and gradual-abrupt (12, 29). In case of abrupt switching, the previous antipsychotics medication is promptly discontinued, while the new one is immediately started (12, 29). The second option is to immediately discontinue the current medication and gradually introducing the new one (abrupt-gradual) (12, 29). In contrast to this option, gradual-abrupt switching involves the gradual dose reduction of the previous medication and the immediate start of the new one (12, 29). Finally, in cross-tapering the new antipsychotic is gradually introduced while the previous is gradually tapered down (12, 29). This can be achieved in two ways as well, either at the same time, or delayed; first, reaching a plateau where the target dose of the new antipsychotic is achieved and only then starting to decrease the dose of the previous medication (full-dose overlap) (12).

**Figure 1:**
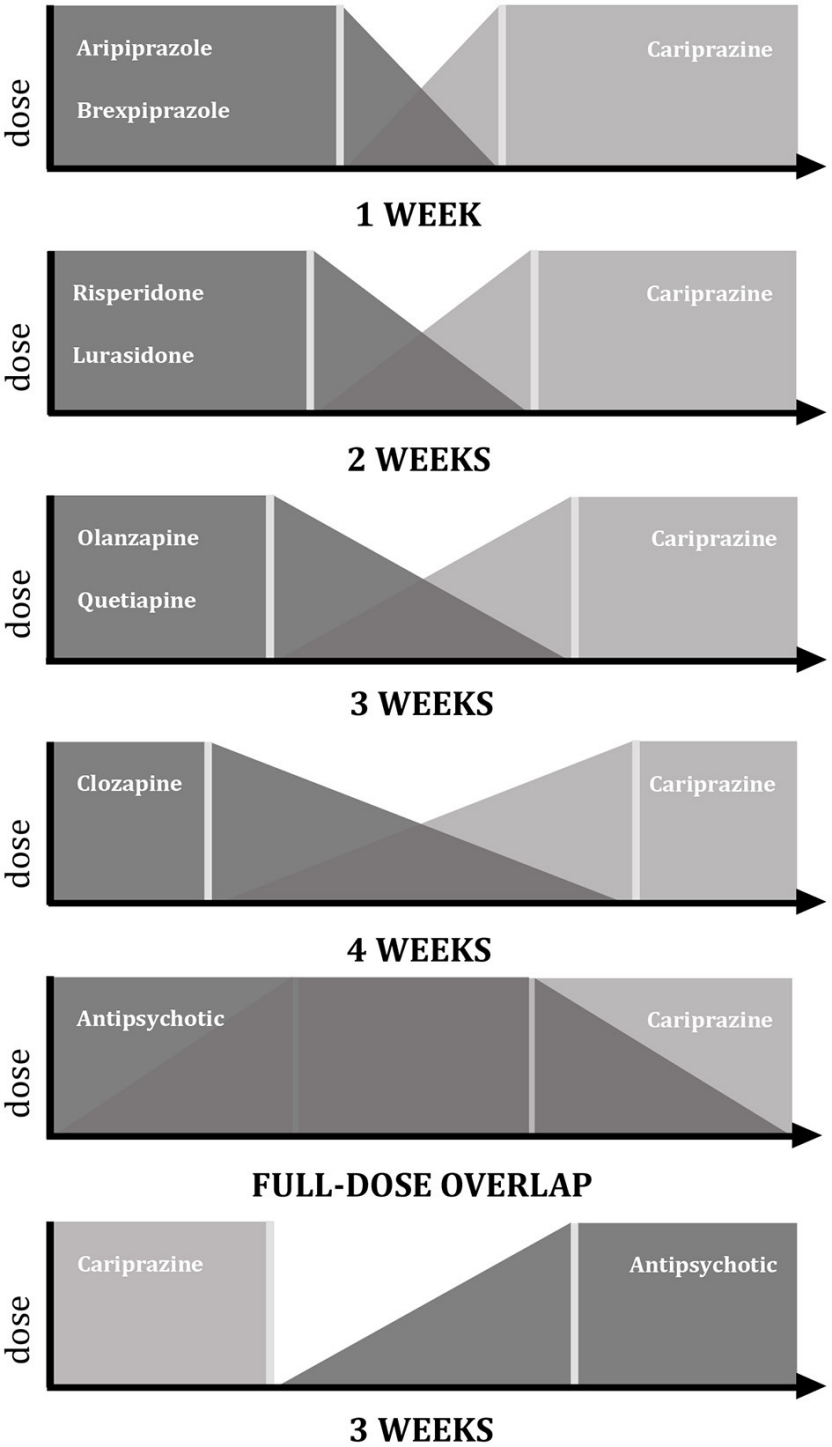
Switching strategies with cariprazine. Graphic representation of data according to Németh et al. (7), Fagiolini et al. (45), Sullivan et al. (46), Stephen (47).

The most recommended strategy for switching to cariprazine is gradual cross-titration with different timeframes depending on the mechanism of action of the previous medication as seen in Figure 1 (29, 48). In case of antipsychotics that have a similar profile to cariprazine i.e., partial agonism at the D2 receptor with comparable histaminergic and cholinergic affinity (e.g., aripiprazole), a 1-week cross-titration is recommended where the previous drug is tapered off within 7 days while at the same time cariprazine dose is escalated to the target dose (29, 48). In contrast, about 2 weeks is necessary if switching from a second-generation antipsychotic that has D2 antagonism (e.g., risperidone) in order to avoid dopaminergic rebound causing increased psychotic symptoms, agitation and dyskinesia (29, 48). Finally, most time (3-4 weeks) should be given when switching from antipsychotics with completely different receptor profiles i.e., those with stronger antihistaminic and/or anticholinergic affinity (e.g., olanzapine, quetiapine or clozapine) so that histaminergic and cholinergic rebound is avoided hence reducing the risk of insomnia, nausea and vomiting (29, 48). At last, various panels emphasize the advantages of a full-dose overlap when switching to cariprazine regardless of the type of antipsychotic drug that has been taken by the patient (45, 46). In such case, a period of overlap for about 2 weeks is recommended before the tapering down of the previous medication hence ensuring that there will be no relapse of symptoms (45, 46).

When analyzing data from clinical cases however, the picture regarding switching strategies is much more mixed. In case of switching from risperidone, some chose abrupt switching (42), while others discontinued it over 2, 9 or 10 days period while gradually increasing the dose of cariprazine (34, 41, 43). Similarly, abrupt stop as well as cross-titration over a 3-day, 2- or 3-week period was described when switching from olanzapine to cariprazine (34, 38, 41). Interestingly, abrupt switching was frequently described in case of quetiapine (36, 41) and clozapine as well (37). In case of making the clinical decision to switch from cariprazine to another antipsychotic, the abrupt-gradual strategy is recommended due to the long half-life of cariprazine (28).

Even if carefully planned and executed however, complications throughout switching can still emerge. In case of a dopaminergic rebound, the re-initiation or dose increase of the previous antipsychotic is recommended (29). If appropriate, this strategy can also be applied to cholinergic and histaminergic rebounds, however in general, the adding of an anticholinergic (e.g., biperiden) or antihistamine (e.g., hydroxyzine) medication can also solve the complications (29). One of the most common side effects of antipsychotic medications is akathisia which can also emerge during a switching period and is recommended to be managed with beta-blockers (e.g., propranolol), benzodiazepines or anticholinergics (29).

### Maintenance Treatment and Concomitant Medications

Maintenance treatment involves the stabilization of the patient on a certain dose that has the ability to control the patient's symptoms without causing any side effects that are intolerable for the patient. Among the four available doses of cariprazine, all doses can be utilized as maintenance dose—depending on the patient's symptom and side effect profile. For instance, although the 1.5 mg/day is most often prescribed in the treatment initiation phase, 11% of the patients in the Latvian study and 4% of the reviewed cases remained on it long-term. Importantly, the rest of the doses were used as maintenance dose equally in these real-life settings; about one fourth of the patients were on 3.0 mg/day, one fourth on 4.5 mg/day and another one fourth on 6.0 mg/day. These data also shows that patients stabilizing on 6.0 mg/day are more usually psychotic and hence require higher D2 activity, while the lower doses found to be more adequate for improving negative and cognitive symptoms. If looking at the pooled data of the fixed-dose studies, based on the effect sizes for the PANSS total and positive symptom factor scores, the optimal dose for most schizophrenia patients is the 4.5 mg/day (49). Thus, prescribing this dose for an adequate time is recommended before switching from cariprazine to another antipsychotic medication due to insufficient effectiveness.

Additionally, the final dose of cariprazine is often related to the maintenance dose of the previous antipsychotic medication (29). Equivalent doses of different antipsychotics are clearly described in The Maudsley Prescribing Guidelines in Psychiatry (50) where it has been stated that 3 mg cariprazine is approximately the same dose as 3 mg risperidone, 10 mg olanzapine, 80 mg lurasidone, 2 mg brexpiprazole, 300 mg quetiapine and 400 mg amisulpride. Indeed, in the negative symptom study, patients were randomized to receive fixed doses of cariprazine (3 mg, 4.5 mg or 6 mg per day) or the equivalent in risperidone (3 mg, 4.0 mg or 6 mg per day) (7). Additionally, the same principle was applied in a case by Di Sciascio et al. that reported a successful switch and maintenance from risperidone 3 mg to cariprazine 3 mg per day (34).

Even though cariprazine is approved for mono-therapeutic use, polypharmacy—essentially the use of more medications—is quite common in real-life settings (51, 52). In fact, in 5 out of the 29 cases was cariprazine used as an add-on treatment (32, 36, 42). To give an example, De Berardis and colleagues utilized cariprazine successfully in combination with clozapine in two patients and reported the effects of cariprazine combination to be remarkable not only regarding symptom control but also concerning the management of side effects caused by clozapine (32). Moreover, in the Latvian observational study, 27% of patients were taking quetiapine, 10% olanzapine and 9% clozapine at their last visit, mostly for non-specific sedation or control of anxiety (27). Importantly, in a *post-hoc* analysis of the cross-titration period of the negative symptom study (7), the co-administration of cariprazine with other antipsychotic medications did not show an unexpected safety profile nor overlapping toxicities, suggesting that it is unlikely that safety will be compromised if polypharmacy with cariprazine is unavoidable (53). This shows that in certain cases patients can benefit from cariprazine combination treatment, nonetheless only if the second antipsychotic is well-chosen with careful consideration regarding the compatibility of the two medications (54).

Concomitant medications other than antipsychotics are well-described in the cariprazine literature. In the clinical trials zolpidem, zaleplon, chloral hydrate, or eszopiclone for insomnia, diphenhydramine, benztropine, or propranolol as rescue medication for extrapyramidal (EPS) symptoms, and lorazepam for agitation, restlessness, irritability, and hostility were permitted (20, 21, 23, 26). Similarly, in the Latvian observational study anti-EPS medication, benzodiazepines, mood stabilizers and antidepressants were allowed (27). Nonetheless, it is important to note that fewer patients needed concomitant medication with cariprazine compared to the antipsychotic they were previously on; 14% of the patients stopped taking anti-EPS medication, 5% antidepressants and mood stabilizers and 3% benzodiazepines (27). In the reviewed cases, most concomitant medications were benzodiazepines (alprazolam, lorazepam and diazepam) (33, 35, 38, 40) and biperiden (36, 38).

## Discussion

The success of antipsychotic treatment depends not only on the mechanism of action of a compound but also on the physician's ability to find the right dosing strategy in which the medication is introduced to the patient. This is especially important, as high levels of non-adherence is caused by issues with ineffectiveness and adverse drug reactions which in turn can increase the risk of relapse (55, 56). With years of practice with a certain antipsychotic medication, clinicians can make confident decisions on how to switch from one medication to another, but this is more complicated with a novel compound such as cariprazine where most data is coming from clinical trials where the conditions are often different from what is seen in real-life practice.

Based on the reviewed literature, it can be stated that evidence regarding dosing, titration and switching strategies with cariprazine is not that different from trials compared to real-life settings. Almost all patients outside of clinical trials received 1.5 mg/day as their first dose of cariprazine, as recommended by the SmPC, however those who started with higher doses tolerated cariprazine just as well and reported effectiveness soon after the beginning of treatment. Higher initial doses might work if they are the corresponding dose of the previous medication or if they are at least half of the target dose.

More variance was found in how cariprazine was up-titrated; compared to the 1.5 mg increase a day or every second day, the dose of cariprazine was increased every third or fourth day, depending on the down-titration of the outgoing antipsychotic medication. Importantly, quicker up-titration was utilized when patients were not switching from another medication but were drug-free or acutely ill with mostly psychotic symptoms. In contrast, slower titration strategies seem to work for patients with more negative symptoms better.

Cross-titration strategies from antipsychotics with different receptor profiles and mechanism of action were also reviewed in detail and evidence shows that gradual switching where the dose of the outgoing antipsychotic is continuously decreased while cariprazine dose is increased is the safest option, as with this strategy the risk of rebounds and adverse reaction are the lowest. The timeframe of the cross-titration should depend on the previous medication; the more similar to cariprazine, the less time is needed. In case of the emergence of any side effects such as anxiety or agitation during the cross-titration period, three options are present—decreasing the dose of cariprazine, slowing down the titration process or control with additional medication such as benzodiazepines or quetiapine.

After the cross-titration period, maintenance treatment follows where patients were found to receive 3.0 mg, 4.5 mg, and 6.0 mg per day equally often in real-life settings. However, when analyzing the clinical data, 4.5 mg/day was reported as the most appropriate dose. Although recommended as monotherapy, cariprazine was also found to be effective in combination with other medications such as clozapine. If polypharmacy is unavoidable however, the compatibility of the drugs in terms of receptor affinity and mechanism of action should be evaluated.

## Data Availability Statement

Data presented in the article can be found in already published materials that are cited accordingly. Further questions should be directed to the corresponding author.

## Author Contributions

ER, ZBD, and ÁB contributed to the conception of the manuscript. ZBD wrote the first draft of the manuscript. All authors contributed to manuscript revision, read, and approved the submitted version.

## Funding

Gedeon Richter Plc. provided funds for the open access publication fees. The funder had no further input in the preparation of this article.

## Conflict of Interest

ZD and ÁB are employees of Gedeon Richter Plc. The remaining author declares that the research was conducted in the absence of any commercial or financial relationships that could be construed as a potential conflict of interest.

## Publisher's Note

All claims expressed in this article are solely those of the authors and do not necessarily represent those of their affiliated organizations, or those of the publisher, the editors and the reviewers. Any product that may be evaluated in this article, or claim that may be made by its manufacturer, is not guaranteed or endorsed by the publisher.
